# The influence of antenatal and pregnancy-related factors on recovery after childbirth: a systematic review

**DOI:** 10.1016/j.xagr.2026.100610

**Published:** 2026-01-27

**Authors:** Zayël Z. Frijmersum, Eva Van der Meij, Ralph De Vries, Johannes R. Anema, Judith A.F. Huirne, Petra C.A.M. Bakker

**Affiliations:** 1 Department of Obstetrics and Gynecology, Amsterdam Reproduction and Development Research Institute, Amsterdam University Medical Center, VU Medical Center (Drs Frijmersum), Amsterdam, The Netherlands; 2 Department of Obstetrics and Gynecology, Amsterdam University Medical Center (van der Meij, De Vries, Anema, Huirne, Bakker), Amsterdam, The Netherlands

**Keywords:** antenatal care, functional ability, maternal health, postpartum recovery, pregnancy-related factors, social participation

## Abstract

**OBJECTIVE:**

This study aimed to identify antenatal and pregnancy-related factors that affect recovery after childbirth, with the purpose of improving maternal health, return to work, and social participation.

**DATA SOURCES:**

We searched PubMed, Embase, and Web of Science up to October 2024 using predefined search terms. The review is registered in PROSPERO (#CRD42022361262).

**STUDY ELIGIBILITY CRITERIA:**

We included cohort studies, randomized controlled trials, and cross-sectional studies published in English or Dutch that evaluated antenatal and pregnancy-related factors associated with maternal morbidity beyond 6 weeks postpartum among women aged ≥18 years who delivered live-born singletons. A minimum follow-up of 6 weeks postpartum was required.

**METHODS:**

Two reviewers independently screened and assessed studies for quality. Data were synthesized narratively, organized by recovery domain.

**RESULTS:**

A total of 56 studies were included. Seven categories of influencing factors were identified: mental health in pregnancy (n=18), demographic and socioeconomic background (n=17), medical and psychological history (n=16), lifestyle in pregnancy (n=12), physical health in pregnancy (n=9), prepregnancy social and lifestyle factors (n=8), and psychosocial resources in pregnancy (n=5). Recovery outcomes included persistent pain, mental health problems, functional ability, and urinary incontinence. Although 47 studies focused on physical or mental health, only 9 studies assessed functional ability. No studies explicitly examined social participation. Previous pain, high body mass index, and antenatal mental health problems were consistently associated with poorer recovery outcomes. In contrast, physical activity, social support, and psychological resilience were generally protective factors.

**CONCLUSION:**

Postpartum recovery is influenced by multiple antenatal and pregnancy-related factors. A personalized, biopsychosocial approach to care may improve maternal health outcomes and social participation.

**AJOG Global Reports at a Glance**

**Why was this study conducted?**

This study aimed to identify which antenatal and pregnancy-related factors influence recovery after childbirth, recognizing that postpartum recovery extends beyond physical healing and includes mental, functional, and social dimensions.

**Key findings**

Mental health problems during pregnancy, preexisting pain, and limited social support were consistently associated with delayed or impaired recovery. Physical activity and strong psychosocial resources during pregnancy were linked to improved recovery outcomes. No studies explicitly evaluated recovery outcomes related to social participation.

**What does this add to what is known?**

This review provides a comprehensive overview of antenatal and pregnancy-related factors affecting recovery after childbirth across multiple domains, using the International Classification of Functioning, Disability and Health framework. Findings highlight the need for a biopsychosocial approach to antenatal and postpartum care to enhance maternal health and social participation.

## Introduction

The postpartum period has traditionally been defined as a 6- to 8-week phase of physical recovery, ending when a woman’s body returns to its prepregnancy state.^1^ However, recovery after childbirth is increasingly recognized as a multidimensional process, affecting women’s physical, psychosocial, and emotional health.

Maternal morbidity is defined as “any health condition attributed to and/or complicating pregnancy, and childbirth that negatively affects well-being and/or functioning.”^2^ “Functioning” in this context extends beyond physical symptoms and includes the ability to engage in employment, leisure, and social participation. Thus, recovery should not only be viewed as symptom resolution, but also as the ability to resume meaningful daily activities and social roles. Maternal morbidity remains a major public health concern, and although its global prevalence is not precisely known, it is believed to be increasing. One reason for this increase in prevalence could be the lack of maternal health–related functioning tools to assess functioning and the broader impact of recovery across the perinatal period.^3^

A previous systematic review has explored delivery-related factors influencing recovery after childbirth.^4^ However, antenatal and individual characteristics may also shape postpartum recovery. Measuring their impact is challenging because postpartum recovery spans multiple domains, and there is no consensus on how to best define or assess it.^5^ In this review, we conceptualize recovery after childbirth using the International Classification of Functioning, Disability and Health (ICF) framework, which emphasizes health, ability, and activity.^6^ We therefore define postpartum recovery as encompassing physical, mental, and sexual recovery, and resumption of everyday activities and social roles.

## Objective

This systematic review aimed to identify antenatal and pregnancy-related factors that influence recovery after childbirth. Our aim is to support early identification of risk factors and guide personalized interventions that improve maternal health, facilitate return to work, and enhance social participation.

## Methods

### Literature review

The review followed PRISMA (Preferred Reporting of Items for Systematic Reviews and Meta-Analyses) guidelines^7^ and was preregistered with PROSPERO (#CRD42022361262). Although the initial title referenced both pregnancy- and delivery-related factors, we subsequently refined the scope to focus exclusively on antenatal and pregnancy-related factors in this review. A first complementary review was conducted on delivery-related factors affecting postpartum recovery to maintain conceptual clarity and reduce heterogeneity.

### Eligibility criteria

We included cohort studies, randomized controlled trials (RCTs), and cross-sectional studies that examined antenatal and pregnancy-related factors influencing postpartum recovery. Studies required a minimum follow-up duration of 6 weeks after delivery and were written in English or Dutch. Studies focusing solely on delivery-related factors were excluded because these were previously addressed in a separate review by our group. Only studies assessing antenatal or pregnancy-related factors and their association with postpartum morbidity were included.

### Participants

Women aged ≥18 years with a live-born singleton were included in this review.

### Affecting factors and interventions

Included studies examined antenatal factors (eg, medical history, mental health, body mass index [BMI]), social factors (eg, social participation, employment), and/or pregnancy-related factors or interventions. Exposure groups (patients exposed to the affecting factor) were compared with the unexposed or control groups.

Recovery outcomes had to be assessed beyond 6 weeks postpartum.

### Outcome measures

Outcome domains were predefined on the basis of the concepts of health, ability, and activity, as defined by the IMB Community Foundation.^6^ These domains included physical, mental, and functional recovery, as well as social participation (Table 1). Although not all domains were equally represented in the included literature, we retained them to capture the breadth of postpartum recovery. Outcomes regarding anatomic changes were not considered for inclusion unless they were explicitly linked to morbidity, functional limitations, or reduced quality of life. This approach aligns with our aim to assess postpartum recovery from a biopsychosocial perspective rather than as isolated physiological outcomes.

**Table 1:** Recovery outcome domains (based on the International Classification of Functioning, Disability and Health framework)

Recovery domain	Description
Physical health	(Preexisting) physical symptoms
Mental health	Mental health problems or complaints
Sexual health	Dyspareunia, sexual functioning
Functional ability	Self-care and resumption of daily activities
Social participation	Return to work, leisure activities, social role functioning

*Frijmersum. Antenatal and pregnancy-related factors of postpartum recovery. Am J Obstet Gynecol Glob Rep 2026*.

### Search strategy

Systematic searches were performed in PubMed, Embase, and Web of Science (Core Collection) up to October 2024. Key words and index terms included “Pregnancy,” “Medical History,” “Complications,” “Functional ability,” “Recovery,” and “Societal participation.” Search strategies for each database are provided in the Appendix. Duplicate removal followed the Amsterdam Efficient Deduplication and Bramer methods using EndNote 20.0.1 (Clarivate, Philadelphia, PA).^8^^,^^9^

### Study selection

Titles and abstracts were screened independently by 2 reviewers (Z.F. and E.V.M.), followed by full-text reading. Disagreements were resolved through a consensus procedure with a third reviewer (P.C.A.M.B.).

### Data extraction

We developed a data extraction form where one reviewer (Z.F.) extracted and a second reviewer (E.V.M.) verified accuracy. Discrepancies were resolved by consulting the third reviewer (P.C.A.M.B.).

### Data items

We extracted data on (1) study characteristics (authors, year, design, sample size, recruitment period), (2) participant characteristics (eligibility, parity, age, interventions), (3) type of antenatal/pregnancy-related factor or intervention, (4) control group, and (5) recovery outcome (type, measurement, timing, follow-up duration).

### Quality assessment

Risk of bias in nonrandomized studies was assessed using the Newcastle–Ottawa scale (NOS) for cohort and cross-sectional designs.^10^ One reviewer (Z.F.) independently judged the quality of the included studies, categorizing them as good-, fair-, or poor-quality on the basis of selection, comparability, and ascertainment of exposure or outcome. According to NOS, quality categories are classified as good, fair, and poor.

### Data synthesis

Because of substantial heterogeneity in study design, number of affecting factors, intervention, control groups, and type of outcome measures, it was not possible to conduct a meta-analysis. Instead, we used a narrative synthesis approach in line with PRISMA recommendations for complex data.^11^ This structured synthesis enabled consistent comparison across studies despite methodological differences.

## Results

### Search results

A total of 11,272 references were identified: 3460 in Ovid Medline, 4059 in Embase, and 3753 in Web of Science. After deduplication, 5448 records remained. The flowchart of the search and selection process is presented in the Figure.

**Figure:**
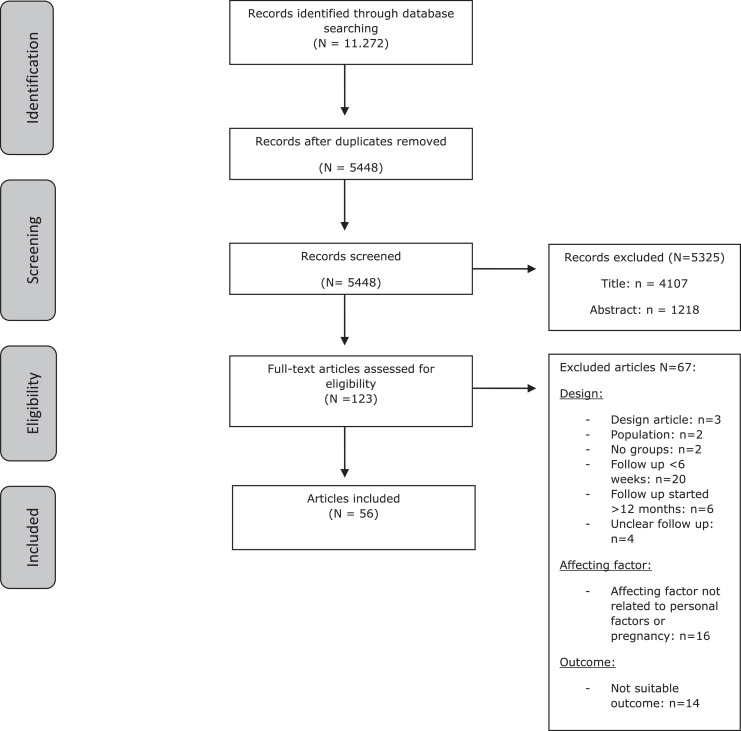
Flowchart of the search and selection procedure of studies

Frijmersum. Antenatal and pregnancy-related factors of postpartum recovery. Am J Obstet Gynecol Glob Rep 2026.

### Study characteristics

Of the 56 included articles, most were cohort studies (n=31; 26 prospective, 5 retrospective). Eleven studies were longitudinal population-based studies, 6 cross-sectional studies, 4 RCTs, 3 case–control studies, and 1 quasi-experimental study. Most studies (n=49) evaluated the relation between specific antenatal and/or pregnancy-related factors (affecting factors) and recovery after childbirth. Nine evaluated the effect of a specific intervention. Studies evaluating an affecting factor compared exposed groups with unexposed groups. For example, when the effect of preexisting pain on recovery after childbirth was evaluated, outcomes were compared between women with preexisting pain and those without preexisting pain. Follow-up ranged from a minimum of 6 weeks to 24 months after childbirth.

### Participants

All participants were aged ≥18 (range, 18–47) years. Most studies (n=43) included both primiparous and multiparous women; 11 focused on primiparous women, and 1 study did not report parity.

### Types of affecting factors and interventions

Seven different categories of antenatal and pregnancy-related factors were identified. First, antenatal factors were categorized into demographic and socioeconomic background, medical and psychological history (previous or preexisting conditions), and prepregnancy social and lifestyle factors. Pregnancy-related factors were categorized into physical health in pregnancy, mental health and emotional state, lifestyle, and psychosocial resources in pregnancy.

### Type of outcome measures

Studies often reported on multiple outcomes. The most frequently reported outcomes were persistent (genitopelvic or postsurgical) pain (n=34), mental health problems (n=14), functional ability (n=9), and urinary incontinence (n=2). These outcome measures were subsequently grouped into broad recovery domains based on the ICF framework, which conceptualizes health as a dynamic interaction between physical, mental, and social components of functioning. In this review, we applied the ICF framework to organize recovery outcomes into 4 domains: physical, mental, functional recovery, and social participation.

We acknowledge that some outcomes might be underrepresented despite their clinical relevance.

### Quality assessment of included studies

Study quality was assessed using the (adapted) NOS for observational studies and the RoB 2 risk-of-bias tool for RCTs (Cochrane, London, United Kingdom). Overall, studies were judged to be of good quality, and the included RCTs showed low risk of bias.

Table 2^12^, ^13^, ^14^, ^15^, ^16^, ^17^, ^18^, ^19^, ^20^, ^21^, ^22^, ^23^, ^24^, ^25^, ^26^, ^27^, ^28^, ^29^, ^30^, ^31^, ^32^, ^33^, ^34^, ^35^, ^36^, ^37^, ^38^, ^39^, ^40^, ^41^, ^42^, ^43^, ^44^, ^45^, ^46^, ^47^, ^48^, ^49^, ^50^, ^51^, ^52^, ^53^, ^54^, ^55^, ^56^, ^57^, ^58^, ^59^, ^60^, ^61^, ^62^, ^63^, ^64^, ^65^, ^66^ presents the characteristics and outcomes of the included studies.

**Table 2:** Study characteristics

Study characteristics		Patients		Intervention group		Control group	Outcome			
ID	Design	Parity	N	Type of affecting factor	Affecting factor category		Type of outcome measure	Follow-up duration	Quality score^a^	Result^b^
Turgut et al,^12^ 1998	Prospective cohort	Primi, multi	88	1. Advanced age, employment, previous pain 2. Current pain	1. Antenatal (demographic, medical history) 2. Pregnancy (physical health)	1. Young age, no heavy work, no previous pain 2. No pain	Persistent pain	6 mo	Good quality	+: Advanced maternal age, high parity, history of back pain and current back pain X: Heavy work before pregnancy
Barrett et al,^13^ 2000	Cross-sectional study	Primi	484	Dyspareunia before pregnancy	Antenatal (medical history)	No dyspareunia	Persistent pain	6 mo	Good quality	+: Previous dyspareunia risk for persistent dyspareunia
Andersson et al, ^14^ 2006	Prospective cohort	Primi, multi	460	1. Marital status, BMI 2. Mental health symptoms in pregnancy	1. Antenatal (demographic, lifestyle) 2. Pregnancy (mental health)	No mental health symptoms	Postpartum depression	6 mo	Good quality	+: More common in women with history of mental disorders, single women, high BMI
Mogren,^15^ 2006	Cross-sectional study	Primi, multi	464	LBP	Pregnancy (physical health)	No pain	Persistent pain	6 mo	Good quality	+: Level and onset of LBP are strong predictors for persistent pain
Mørkved et al,^16^ 2007	RCT	Primi	301	Exercise in pregnancy	Pregnancy (lifestyle)	No physical activity	Persistent pain, functional ability	7 mo	Low risk of bias	+: Risk reduction of persistent pelvic pain, increase in functional ability
Elden et al,^17^ 2008	RCT	Primi, multi	386	Exercise in pregnancy	Pregnancy (lifestyle)	No physical activity	Persistent pain	3 mo	Low risk of bias	+: Fewer positive pain provocation sites
Gutke et al,^18^ 2008	Prospective cohort	Primi, multi	308	1. Advanced age, employment 2. Pain in pregnancy	1. Antenatal (demographic) 2. Pregnancy (physical health)	No pain in pregnancy	Persistent pain	3 mo	Good quality	+: Advanced age, pain in pregnancy, and work dissatisfaction
Mogren,^19^ 2008	Cross-sectional study	Primi, multi	378	Exercise in pregnancy	Pregnancy (lifestyle)	No exercise	Persistent pain	6 mo	Good quality	X: Physical activity does not impact persistent pain
Zaers et al,^20^ 2008	Retrospective cohort	Primi, multi	50	Anxiety in pregnancy	Pregnancy (mental health)	No mental health symptoms	Mental health problems	6 mo	Good quality	+: Anxiety in late pregnancy predictor
Zelkowitz et al,^21^ 2008	Longitudinal study	Primi, multi	106	History of depression, social relationships	Antenatal (medical history, social)	No previous mental health problems	Mental health problems	2 mo	Good quality	+: Prenatal depression and poor adjustment
Aktan,^22^ 2010	Prospective cohort	Primi, multi	177	1. Parity 2. Anxiety in pregnancy	1. Antenatal (medical history) 2. Pregnancy (mental health)	No anxiety	Functional ability	6 wk	Good quality	+: Higher parity, anxiety in pregnancy
Biering et al,^23^ 2010	Case–control study	Primi, multi	4994	Smoking	Antenatal (lifestyle)	No smoking	Persistent pain	18 mo	Good quality	+: Smoking increased risk for persistent pain
Robinson et al,^24^ 2010	Prospective cohort	Primi, multi	326	Preexisting back pain	Antenatal (medical history)	No back pain	Persistent pain	6 wk	Good quality	+: Preexisting pain associated with persistent pain
Biering et al,^25^ 2011	Case–control study	Primi, multi	4920	Increased prepregnancy BMI	Antenatal (lifestyle)	Normal BMI	Persistent pain	18 mo	Good quality	+: Risk of persistent pain increases with high prepregnancy BMI
Olsson et al,^26^ 2012	Prospective cohort	Primi, multi	242	Pain catastrophizing	Pregnancy (mental health)	No pain catastrophizing	1. Persistent pain 2. Functional ability	6 mo	Good quality	+: More persistent pain −: Higher functional ability
Olsson et al,^26^ 2012	Prospective cohort	Primi, multi	273	Health-related QoL, pain catastrophizing, fear avoidance	Pregnancy (mental health)	No catastrophizing	Persistent pain	6 mo	Good quality	+: High catastrophizing and restricted functional ability risk
Stomp-van den Berg et al,^27^ 2012	Prospective cohort	Primi, multi	598	Employment conditions, history of pain	Antenatal (demographic, medical history)	Unemployment, no history of pain	Persistent pain	12 wk	Good quality	+: History of back pain and uncomfortable postures at work
Bjelland et al,^28^ 2013	Longitudinal population-based study	Prim, multi	41.421	Emotional distress in pregnancy	Pregnancy (mental health)	No emotional distress	Persistent pain	6 mo	Good quality	+: Emotional distress is associated with persistent pain
Mukkannavar et al,^29^ 2013	Cross-sectional study	Primi, multi	284	Previous pain, parity	Antenatal (demographic, medical history)	No previous pain	Persistent pain	12 mo	Good quality	+: History of LBP and multiparity risk factors for persistent pain
Dørheim et al,^30^ 2014	Longitudinal study	Primi, multi	2088	Insomnia	Pregnancy (mental health)	No insomnia	Mental health problems	8 wk	Good quality	+: High insomnia scores were associated with postpartum depression
Glowacka et al,^31^ 2014	Prospective cohort	Primi	150	1. Ethnicity 2. Anxiety in pregnancy	1. Antenatal (demographic) 2. Pregnancy (mental health)	No anxiety	Persistent pain	3 mo	Good quality	+: Pain-related anxiety was associated with persistent pain, other nationality
Haakstad and Bø,^32^ 2015	RCT	Primi	105	Exercise in pregnancy	Pregnancy (lifestyle)	No exercise	Persistent pain	8 wk	Low risk of bias	X: No difference between groups
Mannion et al,^33^ 2015	Prospective cohort	Primi, multi	1574	Advanced age, ethnicity, household income, BMI	Antenatal (demographics, lifestyle)		1. Persistent pain 2. Incontinence	12 mo	Good quality	+ Persistent pain: High BMI and lower household income are risk factors for persistent pain + Incontinence: White women, advanced age, and high household income
Bjelland et al,^34^ 2016	Longitudinal population-based study	Primi, multi	20.248	Advanced age, education level, previous pain	Antenatal (demographic, medical history)		Persistent pain	18 mo	Good quality	+: Advanced maternal age, high level of education, and history of pain
Gausel et al,^35^ 2016	Retrospective cohort	Primi, multi	273	1. Advanced age, preexisting pain 2. Pelvic pain and LBP	1. Antenatal (demographic, medical history) 2. Pregnancy (physical health)	No pain	Persistent pain	6 mo	Good quality	+: Advanced maternal age, pelvic pain in pregnancy are predictors for persistent pain
Jin et al,^36^ 2016	Prospective cohort	Primi, multi	527	1. Previous cesarean delivery 2. Depression in pregnancy	1. Antenatal (medical history) 2. Pregnancy (mental health)	No previous cesarean delivery or history of depression	Persistent pain	12 mo	Good quality	+: Women with a previous cesarean delivery or history of pain at higher risk for persistent pain
Kainu et al,^37^ 2016	Retrospective cohort	Primi, multi	1554	Previous pain	Antenatal (medical history)	No previous pain	Persistent pain	12 mo	Good quality	+: Previous pain is a strong predictor
Abdollahi et al,^38^ 2017	Longitudinal population-based study	Unknown	418	Depression	Pregnancy (mental health)	No depression	Mental health problems	12 wk	Good quality	+: Depression in third trimester risk for persistent depressive symptoms
Daly et al,^39^ 2017	Longitudinal population-based study	Primi, multi	98	1. Socioeconomic background 2. Anxiety	1. Antenatal (background) 2. Pregnancy (mental health)	No anxiety	Persistent pain	4 mo	Good quality	+: Social deprivation predictor for persistent pain X: Maternal anxiety not a predictor
Gürkan et al,^40^ 2017	Nonrandomized, post-test-control	Primi	65	Education program	Pregnancy (psychosocial resources)	No education program	1. Functional status 2. Mental health problems	6 mo	Good quality	X: No difference between groups regarding functional status and postpartum depression
Kjeldraad et al,^41^ 2017	Prospective cohort	Primi, multi	92.947	Hyperemesis gravidarum	Pregnancy (physical health)	No hyperemesis gravidarum	Mental health problems	18 mo	Good quality	+: Higher odds for emotional distress postpartum
Munro et al,^42^^2017^	Prospective cohort	Primi	133	Preexisting pain	Antenatal (medical history)	No preexisting pain	Persistent pain	3 mo	Good quality	X: Not a risk factor for persistent pain
Waterlain et al,^43^ 2017	Longitudinal population-based study	Primi	110	Exercise	Pregnancy (lifestyle)	No exercise	Functional ability	2 mo	Good quality	+: Improvement of QoL and increase of physical health
Chortatos et al,^44^ 2018	Prospective cohort	Primi, multi	52.678	Nausea and vomiting	Pregnancy (physical health)	No nausea or vomiting	Persistent pain	6 mo	Good quality	+: Longer duration of nausea and vomiting associated with higher risk for persistent pain
Fathi et al,^45^ 2018	Cross-sectional study	Primi, multi	437	Income	Antenatal (demographic)		Functional ability	8 wk	Good quality	+: Low income associated with functional ability
Wang et al,^46^ 2018	Longitudinal population-based study	Primi, multi	786	1. Advanced age, BMI, previous cesarean delivery 2. Mental health problems	1. Antenatal (demographic, lifestyle, medical history) 2. Pregnancy (mental health)		Persistent pain	12 mo	Good quality	+: Previous cesarean delivery is a risk factor for persistent pain X: Advanced maternal age, BMI, anxiety in pregnancy are not risk factors
Ertman et al,^47^ 2019	Prospective cohort	Primi, multi	1312	Pain	Pregnancy (physical health)	No pain	Mental health problems	8 wk	Good quality	+: Back and pelvic pain associated with higher risk for depression
Galbally et al,^45^ 2019	Prospective cohort	Primi, multi	211	Depression	Pregnancy (mental health)	No depression	Sexual function	12 mo	Good quality	+: Depression associated with lower female sexual function
Ha et al,^49^ 2019	Prospective cohort	Primi, multi	1807	Exercise	Pregnancy (lifestyle)	No exercise	Persistent pain	6 mo	Good quality	+: Physical activity associated with lower odds of persistent LBP
Torres et al,^50^ 2019	Longitudinal population-based study	Primi, multi	165	Financial problems, history of depression	Antenatal (demographic, medical history)	No financial problems, no history of depression	Mental health problems	24 mo	Good quality	−: Full remission of depression most likely in absence of financial problems and with no history of depression
Borges et al,^51^ 2020	Prospective cohort	Primi, multi	621	1. Smoking 2. Anxiety in pregnancy	1. Antenatal (lifestyle) 2. Pregnancy (mental health)	No smoking, no anxiety	Persistent pain	3 mo	Good quality	+: Smoking and anxiety in third trimester increase risk of persistent pain
Matsuda et al,^52^ 2020	Prospective cohort	Primi, multi	88.711	Excessive weight gain in pregnancy	Pregnancy (lifestyle)	Excessive weight gain	Persistent pain	12 mo	Good quality	+: Excessive weight gain
Tavares et al,^53^ 2020	Prospective cohort	Primi, multi	115	1. Advanced age, parity, previous pain 2. Exercise, pain	1. Antenatal (demographic, medical history) 2. Pregnancy (physical health, lifestyle)	No previous pain, no pain in pregnancy, no exercise	Persistent pain	6 mo	Good quality	+: Pain in pregnancy associated with persistent pain X: Advanced age, parity, exercise during pregnancy, previous pain
Wadhwa et al,^54^ 2020	Retrospective cohort	Primi, multi	152	Exercise	Pregnancy (lifestyle)	No exercise	Functional ability	6 wk	Good quality	+: Time to resume household tasks and time to return to employment shorter
Sigurdardottir et al,^55^ 2021	Cross-sectional study	Primi	721	High BMI	Antenatal (lifestyle)	Normal to low BMI	Incontinence	10 wk	Good quality	+: High BMI associated with urinary incontinence
Xiasheng et al,^56^ 2021	Longitudinal population-based study	Primi, multi	264	Employment circumstances, previous pelvic pain, personality traits	Antenatal (demographic, medical history)	No pelvic pain	Persistent pain	24 mo	Good quality	+: Pelvic pain in previous pregnancy, suboptimal work circumstances −: Conscientiousness and extraversion risk factors for regression of pain
Lin et al,^57^ 2022	Prospective cohort	Primi, multi	590	Self-rated anxiety and depression	Pregnancy (mental health)	No anxiety or depression	Mental health problems	6 wk	Good quality	+: Self-rated anxiety and depression associated with postpartum depression
Matsumara et al,^58^ 2022	Prospective cohort	Primi, multi	88.711	Sufficient social support	Pregnancy (psychosocial resources)	Lack of social support	Mental health problems	12 mo	Good quality	+: Good social support prevents postpartum depression
Wu et al,^59^ 2022	Prospective cohort	Primi, multi	982	Diet	Pregnancy (lifestyle)		Persistent pain	12 mo	Good quality	+: More spicy food consumption associated with higher risk of persistent pain
Yetişkin and Dinç Kaya,^60^ 2022	RCT	Primi	60	Exercise	Pregnancy (lifestyle)	No exercise	Persistent pain	6 wk	Low risk of bias	+: Pelvic floor muscle exercise had a positive effect on pain postpartum
Chunmei et al,^61^ 2023	Prospective case–control	Primi	112	Low self-efficacy	Pregnancy (psychosocial resources)	Good self-efficacy	Persistent pain	6 mo	Good quality	+: Low self-efficacy associated with lower odds of regression
Hannon et al,^62^ 2023	Longitudinal population-based study	Primi	1804	Mental health symptoms	Pregnancy (mental health)	No mental health symptoms	Functional ability	12 mo	Good quality	−: No mental health issues associated with better functional ability
Zhuang et al,^63^ 2023	Prospective cohort	Primi, multi	451	Depression, anxiety, lack of social support	Pregnancy (mental health, psychosocial resources)	No depression or anxiety, good social support	Mental health problems	3 mo	Good quality	+: Postpartum depression associated with high anxiety and depression scores, and lack of social support
Li et al,^64^ 2024	Retrospective cohort	Primi, multi	432	Exercise	Pregnancy (lifestyle)	No exercise	Persistent pain	8 wk	Good quality	−: Physical activity a contributing factor to persistent pain
Robinson et al,^65^ 2024	Prospective cohort	Primi, multi	823	Ethnicity	Antenatal (demographic)		Persistent pain	14 wk	Good quality	+: Women from Southeast Asia and the Middle East had higher risk of persistent pain
Tan et al,^66^ 2024	Prospective cohort	Primi, multi	205	Pain and anxiety	Pregnancy (physical health, mental health)	No pain or anxiety	Mental health problems	10 wk	Good quality	+: Pain and anxiety in late pregnancy are predictors for postpartum depression

*BMI*, body mass index; *LBP*, low back pain; *QoL*, quality of life; *RCT*, randomized controlled trial.

a Newcastle–Ottawa quality assessment score: good (5–9), fair (5–7), poor (<5)

b “+” indicates significant effect in favor of the group exposed to the affecting factor (intervention); “−” indicates significant effect in favor of the nonexposed group (control); “X” indicates no significant difference between the groups regarding the affecting factor.

### Affecting factors

All 56 studies reported on ≥1 antenatal or pregnancy-related factors. We grouped these factors into 7 categories. Antenatal factors included demographic and socioeconomic background (n=17), medical and psychological history (n=16), and prepregnancy social and lifestyle factors (n=8). Pregnancy-related factors included mental health in pregnancy (n=18), lifestyle in pregnancy (n=12), physical health in pregnancy (n=9), and psychosocial resources in pregnancy (n=5).

Table 2^12^, ^13^, ^14^, ^15^, ^16^, ^17^, ^18^, ^19^, ^20^, ^21^, ^22^, ^23^, ^24^, ^25^, ^26^, ^27^, ^28^, ^29^, ^30^, ^31^, ^32^, ^33^, ^34^, ^35^, ^36^, ^37^, ^38^, ^39^, ^40^, ^41^, ^42^, ^43^, ^44^, ^45^, ^46^, ^47^, ^48^, ^49^, ^50^, ^51^, ^52^, ^53^, ^54^, ^55^, ^56^, ^57^, ^58^, ^59^, ^60^, ^61^, ^62^, ^63^, ^64^, ^65^, ^66^ summarizes study characteristics and outcomes.

#### Demographic and socioeconomic background

Seventeen studies reported on the effect of demographic and socioeconomic factors on recovery after childbirth, primarily focusing on physical recovery, functional ability, and mental health.

##### Maternal age and parity

Maternal age was assessed by 7 studies. Four reported that advanced maternal age increased the risk of persistent pelvic pain after childbirth (*P*<.05),^12^^,^^18^^,^^34^^,^^35^ whereas 2 other studies did not support this finding (*P*=.093^46^ and *P*=.6^53^). One study found that advanced maternal age increased the risk of urinary incontinence postpartum (*P*=.004).^33^ Parity showed contradicting results: one study found that high parity was a risk factor for persistent pain (*P*<.005),^29^ whereas another study did not support these findings (*P*=.8).^53^ Functional ability was lower among women with high parity in 1 study, although not significantly (*P*=.058).^22^

##### Ethnicity

Three studies found that women from non-White backgrounds were at increased risk of persistent pelvic pain (*P*<.005).^31^^,^^65^ One study also reported increased urinary incontinence (*P*<.001).^33^

##### Employment and working conditions

Employment conditions were assessed in 4 studies. Work dissatisfaction and poor working conditions (eg, no breaks, bad working posture) were associated with persistent pain (*P*<.01).^18^^,^^27^^,^^67^ Physically demanding work before pregnancy was not significantly associated with persistent pain (*P*=.31).^12^

##### Income, education, and social deprivation

Low income was linked to persistent pelvic pain, lower functional ability, and postpartum depression (*P*<.05).^21^^,^^33^^,^^37^^,^^39^ High income was associated with urinary incontinence (*P*=.036).^33^ Finally, 1 study identified a high level of education as a risk factor for persistent pelvic pain (adjusted odds ratio [OR], 0.75; 95% confidence interval [CI], 0.62–0.91).^34^ Social deprivation also predicted persistent pain.^39^

#### Medical and psychological history

Sixteen studies assessed the influence of medical and psychological history on recovery after childbirth, primarily focusing on physical recovery, functional ability, and mental health.

##### Preexisting pain and dyspareunia

Six studies reported that women with preexisting or previous lumbopelvic pain were more likely to have persistent pain after childbirth (*P*<.01).^12^^,^^27^^,^^29^^,^^34^^,^^35^^,^^67^ Another study reported that previous pain elsewhere in the body also predicted persistent pain (*P*<.001).^37^ Two studies reported no significant association between previous pelvic pain and persistent pain (*P*=.20, *P*=.60).^42^^,^^53^ Dyspareunia before pregnancy was found to be a risk factor for persistent dyspareunia after childbirth (3 months postpartum; OR, 2.3; 95% CI, 1.06–5.01; *P*<.05).^13^

##### Previous cesarean delivery

Two studies reported that previous cesarean delivery was associated with chronic postsurgical pain (*P*<.05).^36^^,^^46^

##### Mental health history and personal traits

Four studies examined the relationship between a history of mental health problems and recovery. Two reported that prior depressive episodes increased the risk of postpartum depression (*P*<.005).^21^^,^^50^ Two other studies reported increased risk of persistent pain after childbirth in women with previous depression (*P*<.001)^36^ or neuroticism personality traits (*P*<.001).^67^

#### Prepregnancy social and lifestyle factors

Eight studies evaluated the influence of prepregnancy social and lifestyle factors on physical and mental recovery after childbirth.

##### Body mass index

Three studies reported that a high BMI was associated with increased risk of persistent lumbopelvic pain after childbirth (OR, 1.5; 95% CI, 1.2–2.0^24^; *P*=.018^33^) and urinary incontinence (OR, 1.94; 95% CI, 1.20–3.14; *P*=.007^55^). One study found no significant risk of persistent pain among women with a high BMI (*P*=.634).^46^ Another study showed that the risk of postpartum depression was increased among women with a high BMI (*P*<.005).^14^

##### Smoking

Two studies reported significantly higher risk of persistent pelvic and post–cesarean delivery site pain among women who smoked during pregnancy (OR, 1.2; 95% CI, 1.0–1.4^23^; adjusted relative risk, 2.22; 95% CI, 1.27–3.88; *P*<.05^51^).

##### Social support

Only 1 study assessed the role of social support during pregnancy on postpartum recovery. Although the association between low social support and postpartum depression was not significant (*P*=.155), the direction of the effect suggested a potential risk.^21^

#### Mental health problems in pregnancy

Eighteen studies reported on mental health problems in pregnancy and the effect on recovery across 3 domains: physical recovery, functional ability, and mental health.

##### Anxiety

Five studies found that anxiety was associated with increased risk of postpartum depression (*P*<.05),^14^^,^^20^^,^^57^^,^^63^^,^^66^ and 2 studies reported associations with persistent genitopelvic pain and persistent pain after cesarean delivery (*P*<.05).^31^^,^^51^ Two other studies found no significant association (*P*=.65^39^ and *P*=.31).^44^ Finally, 2 studies found that anxiety led to lower functional ability after childbirth (*P*=.008).^22^^,^^62^

##### Depression

Depression or depressive symptoms during pregnancy predicted postpartum depression in 4 studies (*P*<.05),^14^^,^^38^^,^^57^^,^^63^ and were associated with persistent postsurgical pain (OR, 4.64; 95% CI, 2.078–10.363; *P*<.001).^36^ One study reported that depression was associated with reduced sexual function and general health (*P*<.001).^48^

##### Psychological factors

Two studies examined the impact of pain catastrophizing during pregnancy on postpartum recovery. One study found that higher pain catastrophizing scores in pregnancy were associated with increased risk of persistent lumbopelvic pain postpartum, whereas lower scores were associated with better functional ability (*P*<.05).^26^ The other study reported a similar direction of effect, although it did not use a validated pain catastrophizing scale.^38^ In addition, 1 study found that women with higher emotional distress scores during pregnancy were more likely to experience persistent pelvic pain after childbirth (*P*=.025).^28^ Finally, women with high insomnia scores during pregnancy were more likely to experience postpartum depression (*P*<.0001).^30^

#### Lifestyle in pregnancy

Twelve studies reported on the influence of lifestyle factors during pregnancy on recovery after childbirth, predominantly regarding physical recovery.

##### Physical activity

Eight studies used persistent pain as an outcome measure. Three studies found that women who exercised during pregnancy had a lower risk of persistent pelvic girdle pain postpartum (*P*<.05^17^^,^^49^^,^^60^), whereas 4 other studies reported no significant effect (*P*>.05).^16^^,^^19^^,^^32^^,^^53^ One study reported a contradictory finding, with increased risk of persistent musculoskeletal pain linked to physical activity (*P*=.046).^64^

##### Dietary factors

Two studies reported on dietary factors associated with persistent pain. One study found an association between spicy food consumption and persistent pain after cesarean delivery, whereas another study reported that women with excessive weight gain during pregnancy had higher risk of persistent pelvic girdle pain (*P*<.05).^52^^,^^59^

#### Physical health in pregnancy

Nine studies reported on the effect of physical symptoms during pregnancy on recovery, with outcomes related to physical and mental health.

##### Lumbopelvic pain during pregnancy

Five studies found that women who experienced lumbopelvic pain in pregnancy were significantly more likely to experience persistent pelvic pain after childbirth (*P*<.05).^12^^,^^15^^,^^18^^,^^35^^,^^53^^,^^66^ One study also reported that lumbopelvic pain in pregnancy was associated with higher risk of postpartum depression (*P*<.001).^47^

##### Excessive nausea and vomiting

One study reported that excessive nausea and vomiting during pregnancy was associated with persistent pelvic pain (OR, 2.83; 95% CI, 2.25–3.57^44^), whereas another found a higher risk of postpartum emotional distress (*P*=.001^68^).

#### Psychosocial resources in pregnancy

Five studies examined the role of psychological resources in pregnancy and postpartum recovery.

##### Low self-efficacy

Low self-efficacy was found to be a risk factor for persistent pelvic pain (*P*<.001).^61^

##### Lack of social support

Two studies found that lack of social support in pregnancy is a risk factor for postpartum depression (*P*<.05).^58^^,^^63^ One study reported that poor psychological adjustment during pregnancy is a risk factor for postpartum depression (*P*<.005).^21^

##### Antenatal educational program

One study evaluated the effect of an educational program in pregnancy and found no significant effect on functional ability postpartum (*P*>.05).^40^

### Summary of results

To provide a comprehensive overview of which antenatal and pregnancy-related factors were associated with different domains of postpartum recovery, we constructed a summary table (Table 3). Factors presented in the table were grouped by recovery domain rather than by factor type. Arrows indicate whether a factor was associated with a negative (↑) or positive (↓) impact on recovery outcomes.

**Table 3:** Summary of factors influencing recovery domains

Recovery domain	Affecting factors
Physical recovery	aAdvanced maternal age ↑; Employment ↑; Exercise in pregnancy ↓; High BMI ↑; Low self-efficacy ↑; Pelvic pain in pregnancy ↑; Current physical activity ↓; Previous pain ↑; Smoking ↑; Spicy food consumption ↓
Mental health	Anxiety in pregnancy ↑; Back and pelvic pain ↑; Depression ↑; Financial problems ↑; Good social support ↓; Insomnia ↑; Lack of social support ↑; Low self-efficacy ↑
Sexual health	Depression ↑
Functional ability	Anxiety in pregnancy ↑; Employment conditions ↑; Exercise in pregnancy ↑; Low income ↑; No mental health symptoms ↓
Social participation	No studies reported on social participation as an outcome

↑ = risk factor (associated with worse recovery outcome). ↓ = protective factor (associated with better recovery outcome). Recovery domains are based on the International Classification of Functioning, Disability and Health framework. Affecting factors may be either personal (preexisting or demographic) or pregnancy-related.

*BMI*, body mass index.

a Most studies found a significant effect, although some reported nonsignificant or opposing findings.

## Discussion

### Principal findings

This review identified 7 categories of antenatal and pregnancy-related factors that influence recovery after childbirth: mental health in pregnancy, demographic and socioeconomic background, medical and psychological history, lifestyle in pregnancy, physical health in pregnancy, prepregnancy social and lifestyle factors, and psychosocial resources in pregnancy. Recovery was defined using the ICF framework, encompassing physical, mental, and functional health and activity.

In the 56 included studies, physical recovery was the most frequently assessed outcome domain, including persistent pain (n=34), urinary incontinence (n=1), and general health issues (n=1). Mental health outcomes were reported in 14 studies, and functional ability in 8 studies. No studies assessed recovery outcomes related to social participation, such as return to work, resumption of daily activities or social roles.

Mental health problems during pregnancy, particularly anxiety, depression, and pain catastrophizing, were consistently strong predictors of poorer postpartum outcomes across all recovery domains. Demographic and socioeconomic factors, such as advanced maternal age, non-White ethnicity, low income, and poor employment conditions, were also associated with increased risk of pain and psychological distress. Medical and psychological history, including preexisting pain or depression, was a frequent predictor of persistent postpartum pain. Lifestyle factors in pregnancy showed conflicting results: although some studies found prenatal exercise to be protective, others reported no or even adverse effects on recovery. Physical symptoms in pregnancy, such as lumbopelvic pain, were frequently linked to persistent pain and emotional distress. High BMI and smoking (as a nonspecified binary variable) were associated with not only persistent pain but also urinary incontinence and depressive symptoms. Some evidence suggested that social and psychological resources may influence recovery outcomes, particularly psychosocial well-being, although findings were limited and heterogeneous.

Overall, these findings highlight the importance of a biopsychosocial approach to maternal care, addressing both vulnerabilities and modifiable risk factors, ideally starting before conception and continuing throughout pregnancy.

### Comparison with literature

This systematic review simultaneously assessed both antenatal and pregnancy-related factors associated with postpartum recovery. Several reviews align with our findings. Rosen and Pukall^69^ assessed risk factors for postpartum genitopelvic pain and dyspareunia, and found that a history of chronic pain and psychosocial factors significantly increased the risk, which is similar to our findings on chronic pain and mental health problems. Dai et al^70^ conducted a systematic review and meta-analysis regarding urinary incontinence in postpartum women, and identified advanced maternal age, multiparity, and high BMI as risk factors for postpartum urinary incontinence, which is similar to our results. Postpartum depression was assessed by Gopalan et al.^71^ Factors increasing the risk of postpartum depression in high-risk populations were identified, such as serious mental illness and non-White ethnicity.^72^, ^73^, ^74^ We found similar results in our review. Regarding lifestyle factors, Davenport et al^75^ reported that prenatal exercise decreased the severity of lumbar and pelvic pain postpartum, although the evidence was of low quality. This is partially consistent with our findings: 3 studies reported a protective effect against persistent pain, whereas 4 studies showed no impact. Dachew et al^76^ found that prepregnancy obesity increased the risk of maternal depressive symptoms after childbirth, which is consistent with our findings.

### Interpretation of results

Few studies assessed recovery after childbirth in the domain of functional ability, and those that did used heterogeneous measures, ranging from validated scales (such as the Inventory of Functional Status After Childbirth, Disability Rating Index, 36-Item Short Form Survey [SF-36], Female Sexual Function Index) to resumption of household tasks or return to employment.^16^^,^^22^^,^^26^^,^^40^^,^^43^^,^^45^^,^^48^^,^^54^ One study found that persistent pain was associated with extended sick leave and higher health care utilization 14 months postpartum, highlighting the real-world functional consequences of poor recovery outcomes.^77^ The lack of uniformity reflects the dominance of biomedical perspectives in postpartum research. Despite the small number of studies using functional ability as an outcome measure, several noteworthy findings emerged. First, 3 studies found that prenatal exercise positively influenced functional outcomes.^16^^,^^43^^,^^54^ Conversely, mental health problems during pregnancy, such as anxiety, pain catastrophizing, and depressive symptoms, were consistently associated with diminished functional ability postpartum.^22^^,^^26^^,^^62^^,^^78^ These findings emphasize the importance of early screening and targeted support for psychosocial distress during pregnancy, which may help improve both mental health and functional recovery after childbirth.

The association between demographic and socioeconomic factors and recovery was complex and context-dependent. Advanced maternal age, for example, was frequently identified as a risk factor for persistent pain and incontinence, although findings were inconsistent and definitions varied among studies.^18^^,^^33^, ^34^, ^35^^,^^46^^,^^53^ Most studies used different age thresholds, limiting direct comparability. Some findings, such as those of Turgut et al,^12^ may reflect population-specific factors such as early childbearing and limited access to obstetrical care in their region. In contrast, poor working conditions and financial difficulties were consistently associated with persistent pain, functional limitations, and postpartum depression.^18^^,^^27^^,^^33^^,^^45^^,^^50^^,^^67^ These findings suggest that postpartum recovery is determined by not only biological factors but also broader social determinants of health. Employment context may be especially relevant to long-term recovery and social participation after childbirth.^79^ Incorporating socioeconomic context into postpartum care may help identify women at risk for delayed recovery.

Medical history, particularly preexisting pain, was a consistent and strong predictor of persistent postpartum pain, confirming previous literature.^80^ Regarding mental health problems, our results reaffirm that previous mental health problems increase the risk of mental health problems after childbirth, highlighting the importance of addressing preexisting vulnerabilities in obstetrical care.^71^ Studies on obesity reported an increase in persistent lumbopelvic pain, postpartum depression, and urinary incontinence.^14^^,^^25^^,^^33^^,^^55^ These findings are alarming in the context of the current obesity epidemic, stressing the need for integrated lifestyle and weight management interventions throughout the perinatal period.^81^, ^82^, ^83^ Our findings on prenatal exercise indicate beneficial effects on recovery, including lower risk of persistent pain^17^^,^^49^^,^^60^ and improved functional ability,^16^^,^^43^^,^^54^ underscoring the importance of a healthy lifestyle. One study reported an association between spicy food consumption during pregnancy and persistent postpartum pain.^59^ Although intriguing, this isolated finding requires replication and may reflect cultural or regional dietary patterns rather than a causal relationship.

Although the role of social support during pregnancy is well-recognized in postpartum health literature, only 1 eligible study in this review evaluated its influence on postpartum depression. Although this study did not find a statistically significant association (*P*=.155), the direction of the effect suggested an increased risk. It is possible that this nonsignificant finding reflects limitations in sample size rather than the absence of a true relationship.^21^ This underscores the importance of including social support as a potential modifiable target in biopsychosocial models of postpartum recovery. Additional evidence by Paz-Pascual et al^84^ supports this notion, demonstrating that partner support was significantly associated with mental health and overall postpartum quality of life, reinforcing the value of relational and psychosocial resources in pregnancy.

The antenatal and pregnancy-related factors associated with poor postpartum recovery outcomes, summarized in Table 2,^12^, ^13^, ^14^, ^15^, ^16^, ^17^, ^18^, ^19^, ^20^, ^21^, ^22^, ^23^, ^24^, ^25^, ^26^, ^27^, ^28^, ^29^, ^30^, ^31^, ^32^, ^33^, ^34^, ^35^, ^36^, ^37^, ^38^, ^39^, ^40^, ^41^, ^42^, ^43^, ^44^, ^45^, ^46^, ^47^, ^48^, ^49^, ^50^, ^51^, ^52^, ^53^, ^54^, ^55^, ^56^, ^57^, ^58^, ^59^, ^60^, ^61^, ^62^, ^63^, ^64^, ^65^, ^66^ include several modifiable elements (such as physical activity, social support, and management of mental health symptoms) that should be considered key targets for early intervention during pregnancy.

A personalized biopsychosocial approach to ante- and postpartum care offers an opportunity to address these modifiable factors in a targeted manner. Although social participation was not directly assessed in the included studies, our findings suggest that improving mental health, addressing chronic pain, and supporting women with financial or social stressors could indirectly enhance recovery and social engagement. Tailoring care to individual risk profiles—such as combining prenatal exercise programs with mental health support and workplace adaptations—may optimize both health outcomes and women’s ability to resume work and caregiving roles.

### Strengths and limitations

This review has several strengths. First, it adopts functional ability as a domain for assessing postpartum recovery, providing a comprehensive definition that includes physical, psychological, and social dimensions of health. Previous research often focused on one single outcome, whereas this review provides a more complete understanding. Second, it presents a broad overview of both antenatal and pregnancy-related factors influencing recovery. Combined with our earlier review of delivery-related factors, it offers a more complete overview of influences across the perinatal period.^4^ Third, the review followed the PRISMA guidelines and was preregistered in PROSPERO, ensuring transparency and methodological rigor, although reproducibility of the results may be constrained by study heterogeneity. Some limitations must be acknowledged. The specific aim of the review may have contributed to over- or underreporting of specific factors. Despite broad search terms, some selection bias toward specific factors cannot be excluded. Outcomes related to anatomic changes were included only if they were linked with reported morbidity, which may have resulted in an incomplete representation of such factors. The relative lack of studies on outcomes such as urinary incontinence or social participation highlights gaps in existing literature and limits the depth of synthesis in these domains. Finally, although heterogeneity in study designs, populations, and outcome measures precluded a meta-analysis, this diversity reflects the complex nature of postpartum recovery and supports the use of a narrative synthesis as the most appropriate method.

### Implications for research and practice

Our findings indicate that women with antenatal mental health problems, a history of medical or psychological conditions, or indicators of social disadvantage may be more vulnerable to poor recovery after childbirth. This highlights the potential value of early identification and support during pregnancy, particularly for women presenting with these risk factors. This review also reveals that functional ability remains an underassessed outcome domain in postpartum recovery research, despite its relevance to daily functioning and social engagement. Future studies should assess postpartum recovery as a multidimensional concept, encompassing physical, psychological, and functional aspects. Furthermore, the limited attention to long-term outcomes, particularly those related to social participation such as return to work, suggests a need for more research that captures the broader trajectory of postpartum recovery. Finally, more inclusive research is needed to ensure representation across diverse ethnicity and socioeconomic groups and help shape more individualized and effective postpartum care strategies.

### Conclusion

Recovery after childbirth is influenced by a complex interaction of medical, psychological, social, and lifestyle-related factors. Mental health symptoms during pregnancy and preexisting pain were consistently associated with delayed or impaired recovery. Some evidence suggests that limited social support may contribute to poorer outcomes, although this requires further research. Despite increasing recognition of recovery as a multidimensional concept, most studies still focused on biomedical outcomes. Conflicting findings on demographic and lifestyle factors highlight the need for more nuanced and context-specific research. A personalized, biopsychosocial approach to antenatal and postpartum care is essential to improve maternal health and social participation.

## Declaration of generative AI and AI-assisted technologies in the writing process

Artificial intelligence (ChatGPT, OpenAI, GPT-4, 2025 version) was used to assist in editing and language refinement of the manuscript. The authors reviewed and consented to all content.

## CRediT authorship contribution statement

**Zayël Z. Frijmersum:** Writing – original draft, Methodology, Formal analysis, Conceptualization. **Eva Van der Meij:** Writing – review & editing, Supervision, Formal analysis. **Ralph De Vries:** Methodology, Data curation. **Johannes R. Anema:** Writing – review & editing, Supervision. **Judith A.F. Huirne:** Writing – review & editing, Supervision. **Petra C.A.M. Bakker:** Writing – review & editing, Supervision.

## Appendix Supplementary materials

Image, application 1
