# Supplementary material for: The influence of antenatal and pregnancy-related factors on recovery after childbirth: a systematic review
Source: AJOG Glob Rep. 2026 Jan 27;6(1):100610. doi: 10.1016/j.xagr.2026.100610 (PMC12955133; doi:10.1016/j.xagr.2026.100610)
Supplement: Supplementary file 1 [file mmc1.docx]

**Appendix: Full search strategies for all databases**

**PubMed Session Results (18 Oct 2024)**

| **Search** | **Query** | **Items found** |
| --- | --- | --- |
| **#5** | **#1 AND #2 AND #3 AND #4** | **2,453** |
| **#4** | **"Time Factors"[Mesh] OR "time"[tiab] OR "period"[tiab] OR "periods"[tiab] OR "Postpartum Period"[Mesh:NoExp] OR "postdeliver*"[tiab] OR "post-deliver*"[tiab] OR "postpartum"[tiab] OR "post-partum"[tiab] OR "puerp*"[tiab] OR "after deliver*"[tiab]** | **6,164,417** |
| **#3** | **"Enhanced Recovery After Surgery"[Mesh] OR "Recovery of Function"[Mesh] OR "Convalescence"[Mesh] OR "recover*"[tiab] OR "convalescen*"[tiab] OR "repair*"[tiab] OR "persistent pain"[tiab] OR "chronic pain"[tiab]** | **1,355,620** |
| **#2** | **"Pregnancy Complications"[Mesh:NoExp] OR "Obstetric Labor Complications"[Mesh] OR "Puerperal Disorders"[Mesh] OR "pregnancy complication*"[tiab] OR "labor complication*"[tiab] OR "labour complication*"[tiab] OR "tear"[tiab] OR "tears"[tiab] OR "lacerat*"[tiab] OR "haemorrhage*"[tiab] OR "hemorrhage*"[tiab] OR "excessive bleeding*"[tiab] OR "analges*"[tiab] OR "pain"[tiab] OR "puerp*"[tiab] OR "hindrance*"[tiab]** | **1,384,069** |
| **#1** | **"Delivery, Obstetric"[Mesh] OR "Parturition"[Mesh] OR "parturition"[tiab] OR "parturient*"[tiab] OR "birth*"[tiab] OR "childbirth*"[tiab] OR "vaginal deliver*"[tiab] OR "normal deliver*"[tiab] OR "forceps deliver*"[tiab] OR "cesarea*"[tiab] OR "caesarea*"[tiab] OR "c section"[tiab] OR "c sections"[tiab] OR "postcesarea*"[tiab] OR "postcaesarea*"[tiab] OR "obstetrical extraction*"[tiab] OR "vacuum extraction*"[tiab] OR "ventouse"[tiab] OR "episiotom*"[tiab] OR "fetal version*"[tiab] OR "foetal version*"[tiab]** | **544,589** |

**Ovid Medline Session Results (18 Oct 2024)**

| **Search** | **Query** | **Results** |
| --- | --- | --- |
| #4 | **1 and 2 and 3** | 3,460 |
| #3 | **exp "Quality of Life"/ or exp "Activities of Daily Living"/ or exp "Social Isolation"/ or exp "Social Marginalization"/ or exp "Social Vulnerability"/ or exp "Psychology, Industrial"/ or exp "Work"/ or exp "Employment"/ or ("quality of life" or "life qualit*" or "living qualit*" or "quality of living" or "activities of daily living" or "activity of daily living" or "activities of daily life" or "activity of daily life" or "daily living activit*" or "daily life activit*" or "daily time" or "qol" or "hrql" or "hrqol" or "everyday function*" or "daily function*" or "activities of everyday" or "everyday liv*" or "everyday lif*" or "daily activ*" or "daily task*" or "satisfaction with life" or "life satisfaction" or "societal participation" or "social participation" or "social isolation" or "social marginalization" or "social vulnerability" or "absenteeism" or "presenteeism" or "sick leave" or "labour market" or "labor market" or "work status" or "work participation" or "work engagement" or "work capacity" or "work performance" or job or jobs or employ* or unemploy* or "un employ*" or occupation* or career* or "return to work" or "health status" or "mental abilit*" or "mental capacit*" or "mental status" or "mental health" or "medical history" or "obstetric history" or "pain*" or "incontinen*" or "depress*").ab,ti,kf.** | 3,532,300 |
| #2 | **exp "Enhanced Recovery After Surgery"/ or exp "Recovery of Function"/ or exp "Convalescence"/ or ("convalescen*" or "functional abilit*" or "functional capacit*" or "functional status" or "recover*" or ("pain*" and ("chronic" or "persist*" or "pelvi*"))).ab,ti,kf.** | 1,167,653 |
| #1 | **"Postpartum Period"/ or exp "Puerperal Disorders"/ or ("postdeliver*" or "post-deliver*" or "postpartum" or "post-partum" or "postnatal*" or "post-natal*" or "postcesarean" or "post-cesarean" or "postcaesarean" or "post-caesarean" or "puerp*" or "after cesarean" or "after caesarean" or "following cesarean" or "following caesarean" or "after delivery" or "following deliver*" or "following childbirth" or "following child-birth" or "after childbirth" or "after child-birth").ab,ti,kf.** | 277,344 |

**Embase.com Session Results (18 Oct 2024)**

| **Search** | **Query** | **Results** |
| --- | --- | --- |
| #5 | **#4 NOT ('conference abstract'/it OR 'conference review'/it)** | 4,059 |
| #4 | **#1 AND #2 AND #3** | 7,015 |
| #3 | **'quality of life'/exp OR 'daily life activity'/exp OR 'social isolation'/exp OR 'social exclusion'/exp OR 'social vulnerability'/exp OR 'occupational psychology'/exp OR 'work'/exp OR 'employment'/exp OR ('quality of life' OR 'life qualit*' OR 'living qualit*' OR 'quality of living' OR 'activities of daily living' OR 'activity of daily living' OR 'activities of daily life' OR 'activity of daily life' OR 'daily living activit*' OR 'daily life activit*' OR 'daily time' OR 'qol' OR 'hrql' OR 'hrqol' OR 'everyday function*' OR 'daily function*' OR 'activities of everyday' OR 'everyday liv*' OR 'everyday lif*' OR 'daily activ*' OR 'daily task*' OR 'satisfaction with life' OR 'life satisfaction' OR 'societal participation' OR 'social participation' OR 'social isolation' OR 'social marginalization' OR 'social vulnerability' OR 'absenteeism' OR 'presenteeism' OR 'sick leave' OR 'labour market' OR 'labor market' OR 'work status' OR 'work participation' OR 'work engagement' OR 'work capacity' OR 'work performance' OR 'job' OR 'jobs' OR 'employ*' OR 'unemploy*' OR 'un employ*' OR 'occupation*' OR 'career*' OR 'return to work' OR 'health status' OR 'mental abilit*' OR 'mental capacit*' OR 'mental status' OR 'mental health' OR 'medical history' OR 'obstetric history' OR 'pain*' OR 'incontinen*' OR 'depress*'):ab,ti,kw** | 4,966,221 |
| #2 | **'enhanced recovery after surgery'/exp OR 'convalescence'/exp OR ('convalescen*' OR 'functional abilit*' OR 'functional capacit*' OR 'functional status' OR 'recover*' OR ('pain*' and ('chronic' OR 'persist*' OR 'pelvi*'))):ab,ti,kw** | 1,564,301 |
| #1 | **'puerperium'/exp OR 'puerperal disorder'/exp OR 'postcesarean section'/exp OR ('postdeliver*' OR 'post-deliver*' OR 'postpartum' OR 'post-partum' OR 'postnatal*' OR 'post-natal*' OR 'postcesarean' OR 'post-cesarean' OR 'postcaesarean' OR 'post-caesarean' OR 'puerp*' OR 'after cesarean' OR 'after caesarean' OR 'following cesarean' OR 'following caesarean' OR 'after delivery' OR 'following deliver*' OR 'following childbirth' OR 'following child-birth' OR 'after childbirth' OR 'after child-birth'):ab,ti,kw** | 415,555 |

**Web of Science (Core Collection) Session Results (18 Oct 2024)**

| **Search** | **Query** | **Results** |
| --- | --- | --- |
| #4 | **#1 AND #2 AND #3** | 3,753 |
| #3 | **TS=("quality of life" OR "life qualit*" OR "living qualit*" OR "quality of living" OR "activities of daily living" OR "activity of daily living" OR "activities of daily life" OR "activity of daily life" OR "daily living activit*" OR "daily life activit*" OR "daily time" OR "qol" OR "hrql" OR "hrqol" OR "everyday function*" OR "daily function*" OR "activities of everyday" OR "everyday liv*" OR "everyday lif*" OR "daily activ*" OR "daily task*" OR "satisfaction with life" OR "life satisfaction" OR "societal participation" OR "social participation" OR "social isolation" OR "social marginalization" OR "social vulnerability" OR "absenteeism" OR "presenteeism" OR "sick leave" OR "labour market" OR "labor market" OR "work status" OR "work participation" OR "work engagement" OR "work capacity" OR "work performance" OR "job" OR "jobs" OR "employ*" OR "unemploy*" OR "un employ*" OR "occupation*" OR "career*" OR "return to work" OR "health status" OR "mental abilit*" OR "mental capacit*" OR "mental status" OR "mental health" OR "medical history" OR "obstetric history" OR "pain*" OR "incontinen*" OR "depress*")** | 5,597,750 |
| #2 | **TS=("convalescen*" OR "functional abilit*" OR "functional capacit*" OR "functional status" OR "recover*" OR ("pain*" AND ("chronic" OR "persist*" OR "pelvi*")))** | 1,856,197 |
| #1 | **TS=("postdeliver*" OR "post-deliver*" OR "postpartum" OR "post-partum" OR "postnatal*" OR "post-natal*" OR "postcesarean" OR "post-cesarean" OR "postcaesarean" OR "post-caesarean" OR "puerp*" OR "after cesarean" OR "after caesarean" OR "following cesarean" OR "following caesarean" OR "after delivery" OR "following deliver*" OR "following childbirth" OR "following child-birth" OR "after childbirth" OR "after child-birth")** | 281,343 |
